# Intra-substance steroid injection for full-thickness supraspinatus tendon rupture

**DOI:** 10.1186/s12891-019-2952-y

**Published:** 2019-11-27

**Authors:** Chung-Ting Liu, Ten-Fang Yang

**Affiliations:** 1 Degree Program of Biomedical Science and Engineering, National Chiao Tung University, Hsinchu, Taiwan; 2 Department of Orthopaedics, Mackay Memorial Hospital, Taipei, Taiwan; 3 College of Biological Science and Technology, National Chiao-Tung University, Hsinchu, Taiwan; 4 Graduate Institute of Medical Informatics, Taipei Medical University and Hospital, Taipei, Taiwan

**Keywords:** Steroid, Supraspinatus, Full-thickness, Rupture

## Abstract

**Background:**

The use of steroid injection for treatment of a full-thickness rotator cuff tear is still controversial. This study aimed to evaluate the effectiveness and safety of this treatment method.

**Methods:**

Twelve patients in Group 1 received an intra-substance injection into rupture area of supraspinatus tendon with Diprospan 1 cc (betamethasone disodium phosphate 2 mg and betamethasone dipropionate 5 mg) and 1% xylocaine 1 cc. Twelve patients in Group 2 received an injection with normal saline 1 cc and 1% xylocaine 1 cc. The rupture size was measured by sonography before the injection, 3 months after the injection, and 6 months after the injection. Shoulder Pain and Disability Index (SPADI) score and Pain Visual Analogue Scale (VAS) score were measured and compared between the two groups before the injection, 1 week after the injection, 3 months after the injection, and 6 months after the injection.

**Results:**

Pain and function improved more in Group 1 than in Group 2. The therapeutic effect lasted for at least 6 months in both groups. The size of the supraspinatus tendon rupture was not increased after injection in either group.

**Conclusions:**

Intra-substance injection into rupture area of supraspinatus tendon with steroid and xylocaine is effective to reduce pain and improve function in patients with full-thickness supraspinatus tendon rupture without increasing the size of the rupture.

**Trial registration:**

Current Controlled Trials ChiCTR1900026376, data of registration: 2019/10/05 retrospectively registered.

## Background

Rotator cuff injuries occur most often with repetitive overhead motions in occupational or athletic activities. The risk of rotator cuff injury increases with age. Sometimes, rotator cuff tears may occur due to a single injury.

If activities that aggravate a rotator cuff tear continue despite increasing pain, further damage may occur. A rotator cuff tear can get larger over time. The goal of any treatment is to reduce pain and restore function. Nonsurgical treatment options may include rest, activity modification, nonsteroidal anti-inflammatory drugs (NSAIDs), strengthening exercises, physical therapy, or steroid injection. The chief advantage of nonsurgical treatment is avoidance of surgical risks including infection, post-operative stiffness, lengthy recovery time, and anesthesia complications. Continuing pain is the main indication for surgery. If conservative treatment for the torn rotator cuff fails, surgical repair or joint replacement may be required.

Inflammatory cytokines including tumor necrosis factor α, interleukin 1β, and interleukin 6 are released from the ruptured rotator cuff and these cytokines can be repressed by steroid. However, steroid injection for the treatment of full-thickness rotator cuff tear is still controversial. In-vitro studies have shown steroids have necrosis-inducing effects on fibroblasts and tenocytes [1]. Steroids can cause reduction in the cellular capacity for tendon repair and changes in cellular differentiation [2]. Some animal model studies of rotator cuff tear showed that a single dose of steroid injection significantly weakens injured rat rotator cuff tendons in the acute phase, but this effect is transient as the biomechanical properties returned to control levels [3]. However, repeated steroid injections may damage rat rotator cuffs and potentially harm tendon cells [4, 5].

To date, no research has explored whether the size of a full-thickness rotator cuff rupture increases after a steroid injection. Ultrasound is an accurate, low-cost, and radiation-free modality established for evaluation of rotator cuff tears [6]. This study used ultrasound to measure rotator cuff rupture size before and after the steroid injection. Pain and functional scores were also evaluated before and after the injection. The objective of this study is to evaluate the effectiveness and safety of intra-substance injection into rupture area of supraspinatus tendon.

## Methods

**Fig. 1:**
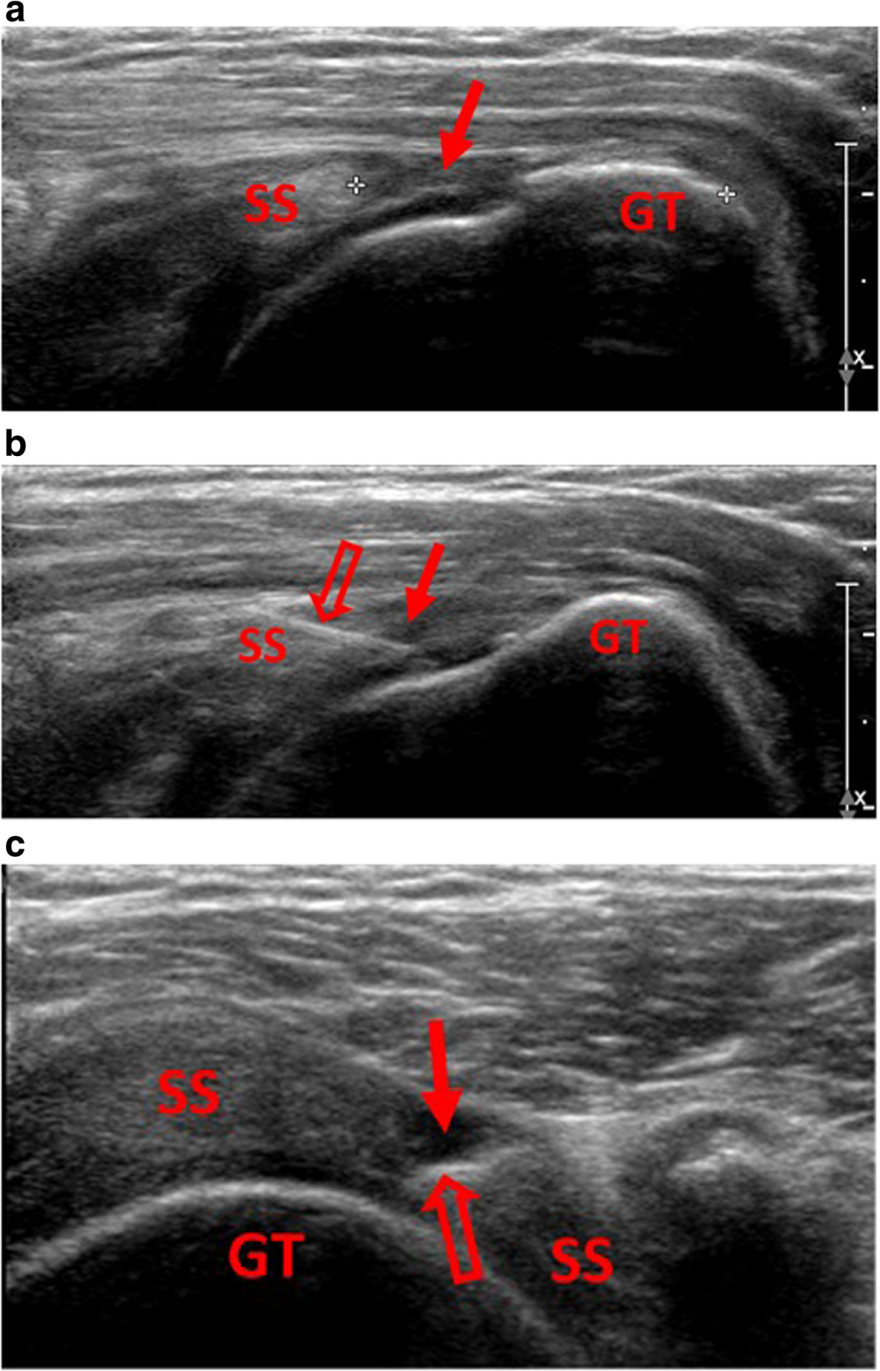
**a** Rupture size in longitudinal axis of supraspinatus tendon was measured by sonography. Hypoechoic area (solid arrow) indicates the rupture area of tendon. **1b** Intra-substance injection was done by echo-guided injection directly into the rupture area (solid arrow) of supraspinatus tendon. Injection needle (hollow arrow) can be clearly seen on sonography in longitudinal view. **1c** Injection needle tip (hollow arrow) can be seen in rupture area (solid arrow) of supraspinatus tendon in transverse view SS = supraspinatus tendon, GT = greater tuberosity of humerus.

This study was approved by the ethics committee of our hospital, and informed consent was obtained from all patients. The principles and laws of clinical trials were followed. We enrolled patients recently diagnosed with a supraspinatus tendon full-thickness rupture between 2017 and 2018. The ruptures which measured between 1 and 3 cm in the longitudinal axis using sonography were included in the study. Exclusion criteria included patients who preferred surgical repair, patients who had a rupture size greater than 3 cm in the longitudinal axis, and patients unable to adhere to shoulder activity restrictions. The enrolled patients were divided into two groups by block randomization. Patients in Group 1 received an intra-substance injection into rupture area of supraspinatus tendon with Diprospan 1 cc (which contains betamethasone disodium phosphate 2 mg and betamethasone dipropionate 5 mg) and 1% xylocaine 1 cc. Patients in Group 2 received an injection with normal saline 1 cc and 1% xylocaine 1 cc. Intra-substance injection into rupture area of supraspinatus tendon was done by echo-guide (Fig. 1a, b, c). The injection fluid will be infused into the subacromial bursa as well as the glenohumeral joint.

Sonography was performed and the size of rupture was measured before the injection, and at 3 and 6 months afterward. All sonography examinations were performed by a single experienced doctor, and all injections were echo-guided. This was a single blind study in which patients were not aware of the injection regimen they received.

Shoulder Pain and Disability Index (SPADI) [7] score and Pain Visual Analogue Scale (VAS) score were measured and compared between the groups before the injection, and at 1 week, 3 months, and 6 months afterward. SPADI is a standardized 13-item questionnaire that elicits information on pain and functional limitations with activities of daily living. The composite SPADI score ranges from 0 to 100, with higher scores reflecting worse pain and function.

In Group 1, fifteen patients were enrolled but 3 were lost during follow-up. The average age was 66.25 years old (range 58 to 73); the average size of the full thickness supraspinatus tendon rupture was 1.89 cm (range 1.0 to 2.8 cm). In Group 2, fifteen patients were enrolled but 2 were lost during follow-up and 1 decided on surgical repair 1 month after the injection. Thus, there were 12 patients for analysis in each group. In Group 2 the average age was 66.33 years old (range 58 to 72); the average size of the full thickness supraspinatus tendon rupture was 2.01 cm (range 1.2 to 2.8 cm).

## Results

### Before the injection

There was no significant difference between the two groups in rupture size, SPADI pain score, SPADI disability score, SPADI total score, or pain VAS before the injection. In Group 1 the average rupture size of the supraspinatus tendon was 1.89 cm (range 1.0 to 2.8 cm), the average SPADI pain score was 61.50 (range 50 to 74), the average SPADI disability score was 43.50 (range 28 to 59), the average SPADI total score was 50.33 (range 28 to 59), and average pain VAS was 8.08 (range 7 to 9). In Group 2 the average rupture size of the supraspinatus tendon was 2.03 cm (range 1.3 to 2.8 cm), the average SPADI pain score was 59.33 (range 50 to 72), the average SPADI disability score was 42.58 (range 34 to 50), the average SPADI total score was 49.00 (range 40 to 58) and average pain VAS was 7.67 (range 7 to 9).

### One week after the injection

The groups exhibited significant differences in the SPADI pain, disability and totals scores, and pain VAS scores, at 1 week after the injection (*p* < 0.001). In Group 1 the average SPADI pain score was 34.00 (range 28 to 52), the average SPADI disability score was 26.83 (range 19 to 41), the average SPADI total score was 29.42 (range 21 to 45), and the average pain VAS was 4.00 (range 3 to 6). In Group 2 the average SPADI pain score was 49.33 (range 42 to 62), the average SPADI disability score was 39.00 (range 30 to 49), the average SPADI total score was 42.67 (range 35 to 54), and the average pain VAS was 6.17 (range 5 to 8).

### Three months after the injection

The groups continued to exhibit significant differences in the SPADI pain, disability and totals scores, and pain VAS scores, at 3 months after the injection (*p* < 0.001). In Group 1 the average SPADI pain score was 38.67 (range 20 to 54), the average SPADI disability score was 28.53 (range 19 to 46), the average SPADI total score was 32.42 (range 20 to 51), and the average pain VAS was 5.00 (range 3 to 7). In Group 2 the average SPADI pain score was 55.50 (range 46 to 64), the average SPADI disability score was 40.92 (range 33 to 48), the average SPADI total score was (range 39 to 54), and the average pain VAS was 7.17 (range 6 to 8).

### Six months after the injection

The groups still exhibited significant differences in the SPADI pain (*p* = 0.0015), disability (*p* = 0.011), and total (*p* = 0.008) scores, and pain VAS scores (*p* = 0.012), at 6 months after the injection. In Group 1 the average SPADI pain score was 45.50 (range 32 to 66), the average SPADI disability score was 31.67 (range 19 to 51), the average SPADI total score was 37.00 (range 26 to 57), and the average pain VAS was 5.92 (range 3 to 8). In Group 2 the average SPADI pain score was 56.17 (range 46 to 66), the average SPADI disability score was 41.00 (range 31 to 49), the average SPADI total score was 46.75 (range 38 to 52), and the average pain VAS was 7.33 (range 6 to 8).

### Rupture size

In Group 1, the average size of the supraspinatus tendon rupture was 1.89 cm (range 1.0 to 2.8 cm) before the injection. The average size was 1.96 cm (range 1.0 to 2.8 cm) and 2.01 cm (range 1.3 to 2.8 cm) at 3 and 6 months after the injection, respectively. There was no significant difference between the rupture size before the injection and at 3 months (*p* = 0.191) or 6 months (*p* = 0.066) afterward.

In Group 2, the average size of the supraspinatus tendon rupture was 2.03 cm (range 1.3 to 2.8 cm) before the injection. The average size was 2.08 cm (range 1.4 to 2.7 cm) and 2.15 cm (range 1.7 to 2.7 cm) at 3 and 6 months after the injection, respectively. There was no significant difference between the rupture size before the injection and at 3 months (*p* = 0.273) or at 6 months (*p* = 0.073) afterward.

### Results summary

The size of the rupture did not increase after intra-substance injection into rupture area of supraspinatus tendon with Diprospan 1 cc and 1% xylocaine 1 cc or injection with normal saline 1 cc and 1% xylocaine 1 cc. In both groups, pain and function significantly improved after the injection. The pain and function scores both improved more in Group 1 than in Group 2. The therapeutic effect lasted for at least 6 months in both groups. (Tables 1 and 2).

**Table 1:** Average values and standard deviations in group 1 and group 2

	Age	RS BI	RS 3 M	RS 6 M	Pain BI	Pain 1 W	Pain 3 M	Pain 6 M	Dis BI	Dis 1 W	Dis 3 M	Dis 6 M	Total BI	Total 1 W	Total 3 M	Total 6 M	VAS BI	VAS 1 W	VAS 3 M	VAS 6 M
Ave in group1	66.25	1.89	1.96	2.01	61.5	34	38.67	45.5	43.5	26.83	28.58	31.67	50.33	29.42	32.42	37	8.08	4	5	5.92
SD in group1	4.42	0.63	0.62	0.56	7.02	7.57	9.96	11.45	9.90	6.30	7.34	9.33	8.39	6.66	8.18	10.05	0.83	0.88	1.04	1.38
Ave in group2	66.33	2.03	2.08	2.15	59.33	49.33	55.5	56.17	42.58	39	40.92	41	49	42.67	46.25	46.75	7.67	6.17	7.17	7.33
SD in group2	4.01	0.52	0.36	0.40	5.45	4.53	5.66	5.84	5.81	5.19	4.48	5.20	5.23	4.56	4.44	4.74	0.72	0.66	0.86	0.91

*Ave* average, *SD* standard deviation, *RS* rupture size, *Pain* SPADI pain score, *Dis* SPADI disability score, *Total* SPADI total score, *VAS* pain VAS score, *BI* before injection, *1 W* 1 week after injection, *3 M* 3 months after injection, *6 M* 6 months after injection

The size of the supraspinatus tendon rupture did not increase in both groups. In both groups, pain and function significantly improved after the injection. The pain and function scores both improved more in Group 1 than in Group 2

**Table 2:**
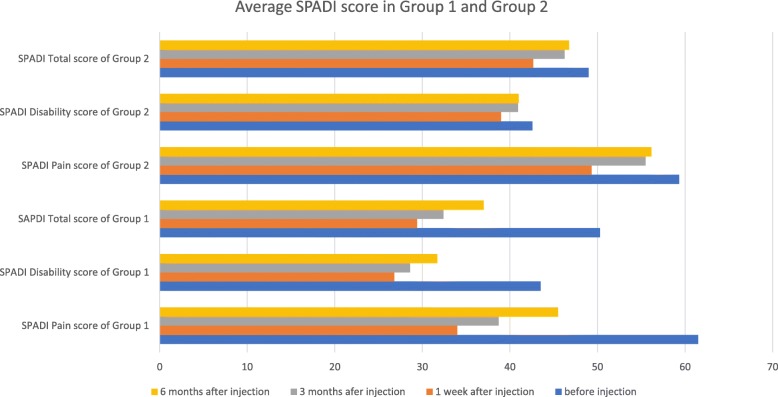
Average SPADI score in Group 1 and Group 2

In both groups, pain and function significantly improved after the injection. Dramatic improvement was noted in group 1 for 6 months. In group2, only slight improvement was **noted** after 3 months.

## Discussion

In the current study, we found that intra-substance injection into rupture area of supraspinatus tendon is effective for pain reduction and shoulder function improvement. However, this conservative treatment option remains controversial. While many studies support the use of steroid injections, there are also many studies that oppose it.

There is a lot of research supporting steroid injection for treatment of rotator cuff symptoms. Cook et al. revealed that steroid injection has a short-term benefit lasting up to 8 weeks, which is better than local anesthetic injections alone; however, no difference was observed beyond 8 weeks [8]. Hart revealed that steroid injection was superior to placebo to improve pain and function only in the short-term [9]. However, Arroll et al. showed that steroid injection can be effective for up to 9 months [10]. In our study, the therapeutic effect of a single steroid injection for rotator cuff rupture lasted at least 6 months. There was significantly better pain relief and functional improvement in comparison to an anesthesia injection.

Steroid injection is also safe for treating rotator cuff problems other than rupture. Chavez-Lopez et al. found that an ultrasound-guided steroid injection has a rapid and sustained overall response for shoulder pain [11]. Yamaguchi et al. found that pain improved with either a steroid or a hyaluronic acid injection compared to saline. Both hyaluronic acid and steroid injections can suppress inflammation [12]. According to Bhatia et al., it was also useful for subacromial impingement syndrome and it was not a causative factor in rotator cuff tear [13]. Fast pain relief with steroid injection was also found in the study by Garg et al. for rotator cuff tendonitis [14].

According to the literature, steroid injection can also be used after a rotator cuff repair. Perdreau et al. found steroid injection after a rotator cuff tear surgery provided immediate benefit in terms of both analgesia and morphine sparing, without apparent risk [15]. Kim et al. also found it effective for shoulder stiffness, pain, and range of motion after arthroscopic rotator cuff repair, without compromising repair integrity postoperatively [16].

The risks of steroid injection for rotator cuff rupture have been evaluated in animal studies. Wei et al. analyzed the type-III to type-I collagen expression ratio and used a tendon injury marker to examine the effects of steroid on an injured rotator cuff in rats. They found a single dose of steroid does not damage the structure of the injured rotator cuff tendon in rats [17]. Another study conducted by Mikolyzk et al. revealed a single dose of steroid weakens both intact and injured rat rotator cuff tendons within 1 week, but this effect is transient as the biomechanical properties of the steroid-exposed groups returned to control levels within 3 weeks [3]. In another animal study conducted by Lee et al. found steroid injection may alter the collagen composition and extracellular matrix, thereby interfering with the healing process of rat rotator cuff tear in the early phase after the injection. However, these alterations seem to become normalized after the early inflammatory healing phase [18]. Therefore, according to these animal studies, the negative effects of steroid injection for rotator cuff rupture do exist, but they are only transient and have no long-term disadvantages.

However, some laboratory research has opposed the use of steroid injection for treating rotator cuff problems. Zhang et al. found necrotic and apoptotic effects of steroids on fibroblasts and tenocytes [1]. Tempfer et al. also found that isolated human rotator cuff tendon cells in vitro showed reduction in the cellular capacity for tendon repair and changes in cellular differentiation after steroid injection [2]. Dean et al. observed rotator cuff cells after surgical repair. The expected increases in cell proliferation, vascularity and HIF-1alpha were not seen after steroid injection. The increase in the glutamate receptor NMDAR1 after steroid injection raises concerns about potential excitotoxic tendon damage [19].

Some animal studies do not support the use of steroid injection for rotator cuff injury, either. In the research of Tillander et al., there was focal inflammation, necrosis, and fragmentation of collagen bundles in rat tendons after 5 steroid injections [4]. Maman et al. also found repeated doses of steroid significantly weakened rats’ rotator cuffs and negatively affected bone quality, in addition to possibly causing deterioration of the osteotendinous junction [20]. In the study by Akpinar et al., fragmentation of collagen bundles and inflammatory cell infiltration were evident after steroid injection. Repeated injection can cause deleterious changes in the normal structure of the rat rotator cuff [5]. However, those data retrieved from animal studies must be scrupulously analyzed before extrapolation to the human population.

Because of concern about the side effect of steroids, there is a lot of research about anabolic steroids. The addition of platelet rich plasma (PRP) does not interfere with the anti-inflammatory effects of a steroid on IL-1beta-treated tenocytes from rotator cuff tears, and it avoids the deleterious side effects of a corticosteroid [21]. Cell viability did not decrease when PRP was injected along with steroid. The number of apoptotic cells increased with steroid use, but addition of PRP prevented cell apoptosis. The deleterious effects of steroid use were prevented by PRP, which can be used as a protective agent for patients receiving local steroid injections [22]. In a sheep model study on rotator cuff tendon tear, further muscle atrophy was prevented with the application of anabolic steroids starting immediately after tendon repair [23]. Anabolic steroids have been shown to be effective in preventing classic degenerative muscle changes after tendon tears [24, 25]. More clinical studies and evidence may be needed to prove the effectiveness of steroid combined with PRP injection for rotator cuff rupture.

Inflammatory cytokines including tumor necrosis factor α, interleukin 1β, and interleukin 6 are released from the ruptured rotator cuff and these cytokines can be repressed by steroid. In the present study, we used echo-guided injection directly into the rupture area of supraspinatus tendon so that the steroid can contribute the anti-inflammation effect on the ruptured tendon immediately. Among the local anesthesia medications, long-acting type bupivacaine is the most toxic for chondrocyte and short-acting type lidocaine used in the current study has less negative effect on it. Bupivacaine should be avoided for intra-articular injection [26].

There were some limitations in our study. Using sonography to measure the rupture size of a rotator cuff can have some error, including inter-observer and intra-observer variability. All sonography examinations in our study were performed by the same experienced doctor to reduce inter-observer variability, but intra-observer variability may still exist. Additionally, our sample size was small, and the duration of follow-up was too short. And there is a measurable increase in size of the defect over the time frame of this study. Although the size of the full-thickness rotator cuff rupture will progress over time in most patients, the long-term clinical effect of repeated injection of steroid for rotator cuff rupture should be investigated in future studies. A larger sample size is also required.

## Conclusion

Intra-substance injection into rupture area of supraspinatus tendon with Diprospan 1 cc and 1% xylocaine 1 cc effectively reduces pain and improves function in patients with full-thickness supraspinatus tendon rupture, without increasing the rupture size. The therapeutic effect lasts at least 6 months and is better than injection with 1% xylocaine alone.

## Data Availability

The datasets used and analysed during the current study are available from the corresponding author on reasonable request.

## Abbreviations

NSAID: Nonsteroidal anti-inflammatory drugs
PRP: Platelet rich plasma
SPADI: Shoulder Pain and Disability Index score
VAS: Pain Visual Analogue Scale
